# Clinical Evaluation of MyoCare in Europe (CEME): study protocol for a prospective, multicenter, randomized, double-blinded, and controlled clinical trial

**DOI:** 10.1186/s13063-023-07696-0

**Published:** 2023-10-17

**Authors:** Cristina Alvarez-Peregrina, Miguel Angel Sanchez-Tena, Clara Martinez-Perez, Cesar Villa-Collar, Clinical Evaluation of MyoCare in Europe –the CEME Study Group, Alejandro Montero-Torrejon, Alfredo Lopez-Muñoz, Alicia Ruiz-Hernandez, Ana Isabel Gonzalez-Abad, Antonio Manuel Santos-de-Melo, Beatriz de Corcuera Terrero, Beatriz Gargallo-Martinez, Carolina Mataix-Palao, Christina Boeck-Maier, Diego Asensio Celdran-Vivancos, Isabel Rodriguez, Javier Vega-Dominguez, João Manuel Martinho-Antunes, Jose Carlos Garay-Dominguez, Jose Ignacio Recalde-Zurita, Juan Luis Reina-Gallego, Laura Rocha, Manuel Lérida, Mariano Gonzalez-Perez, Patricia Silva-Carrola, Paula Alves-Silva, Ramon Gutierrez-Ortega, Raquel Blanco-Cotovio, Raul Manuel Maia, Siegfried Wahl, Timo Kratzer, Vladimiro Oliveira-Hipólito, Arne Ohlendorf

**Affiliations:** 1 Optometry and Vision Department, Faculty of Optics and Optometry, Complutense University of Madrid, Madrid, Spain; 2 ISEC LISBOA, Instituto Superior de Educação E Ciências, Lisbon, Portugal; 3 Faculty of Biomedical and Health Science, European University of Madrid, Madrid, Spain; 4 ZEISS Group, Carl Zeiss Vision International GmbH, Turnstrasse 27, 73430 Aalen, Germany

**Keywords:** Myopia, Progression, Axial length, Spectacle lenses, Myopia control, Clinical trial, European children

## Abstract

**Background:**

Myopia prevalence has been increasing in the last decades, and its pathological consequences, including myopic maculopathy and high myopia-associated optic neuropathy, are now one of the most common causes of visual impairment. It is estimated that by 2050, more than 50% of Europeans and Americans will be myopes, which is alarming due to the high morbidity of myopes over − 6.00D. Once myopia has appeared, there are different options with scientific evidence to try to slow the axial length growth. Ophthalmic lenses are the less invasive treatment to control myopia, and there is evidence about the efficacy of different designs, mainly in the Asiatic population. However, new designs have been launched, and it is not known if efficacy is the same between Asiatic and European subjects. Thus, we have set up a randomized, controlled, double-blind, and multicenter trial to investigate the efficacy of a new design of ophthalmic lenses for myopia control in European children.

**Methods:**

A 2-year prospective, multicenter, randomized controlled, and double-blind clinical trial is used to investigate the efficacy of a new design of ophthalmic lenses to slow the progression of myopia. Three hundred children aged from 6 to 13 years old will be recruited and randomly assigned to a study or control group. The study group will be composed of 150 children wearing MyoCare while the control group will be composed of 150 children wearing Clearview. The inclusion criteria will be myopia with a spherical equivalent between − 0.75D and − 5.00D, astigmatism < 1.50D, and anisometropia < 1.00D and having a historical evolution of at least − 0.50 The primary outcome is to compare the mean annual progression of the spherical equivalent between both groups. The secondary outcomes are axial length, choroidal thickness, phorias, and accommodative status of both groups.

**Discussion:**

This study will be the first randomized and controlled clinical trial in European children with spectacle lenses based on simultaneous competing defocus. The results will shed light on the clinical evidence of spectacle lenses relying on this new design for the management of myopia with results of efficacy in the non-Asiatic population.

**Trial registration:**

EU Clinical Trials Register (EudraCT) 2022–001696. Registered on 27 April 2022. ClinicalTrials.gov NCT05919654. Registered on 26 June 2023.

## Administrative information

**Table:** 

Title {1}	Clinical efficacy of Ophthalmic lenses with ZEISS ClearFocus design (MyoCare) in 6 to 13 years old children: study protocol for a prospective, multicenter, randomized, double-blinded and controlled clinical trial
Trial registration {2a and 2b}	EU Clinical Trials Register, EudraCT: 2022–001696. Registered on 27 April 2022 ClinicalTrials.gov, ID NCT05919654. Registered on 26 June 2023 https://clinicaltrials.gov/study/NCT05919654
Protocol version {3}	V3,24 November 2022
Funding {4}	Carl Zeiss Vision International GmbH supports all the expenses of the clinical trials and provides the ophthalmic lenses
Author details {5a}	Complutense University of Madrid, Faculty of Optics and Optometry, Optometry and Vision Department. Madrid, Spain; ISEC LISBOA (Instituto Superior de Educação e Ciências). Lisbon, Portugal; Clínica Oftalmológica Novovision, Madrid, Spain Carl Zeiss Vision International GmbH, ZEISS Group, Turnstrasse 27, 73,430 Aalen, Germany
Name and contact information for the trial sponsor {5b}	Complutense University of Madrid, Faculty of Optics and Optometry, Optometry and Vision Department. Madrid, Spain Cristina_alvarez@ucm.es + 34913946847
Role of sponsor {5c}	The sponsor has led the study design; collection, management, analysis, and interpretation of the data; writing of the report; and the decision to submit the report for publication

## Introduction

### Background and rationale {6a}

The prevalence of myopia has been increasing in recent decades, and its pathological consequences, including myopic maculopathy and high myopia-associated optic neuropathy, are now one of the most common causes of visual impairment [1]. It is estimated that by 2050, more than 50% of Europeans and Americans and around 90% of the inhabitants of Southeast Asia will be myopic. This is alarming data due to the high morbidity of myopes who have more than − 6.00D [2].

Due to the previously exposed data, establishing strategies to prevent the onset of myopia and its progression in terms of the refraction (spherical equivalent refractive error in diopter (D) (SE) and the length of the eye (axial length in mm (mm) (AxL)) to high myopia (SE <  − 6D or > 26 mm) is highly recommended. Thus, an effective clinical intervention at an early age will reduce the further progression of myopia and inhibit the complications that are associated with myopia and especially high levels myopia, such as the said retinal pathologies [3].

There is already clinical and real-world evidence about effective actions and interventions to reduce the progression of myopia in terms of SE and AxL [4].

Once myopia has appeared, there are different techniques with clinical and scientific evidence to manage the progression of myopia (for both SE and AxL). Among the different techniques for the control of myopia, the following interventions are available [3, 5]:

1. Time spent outdoors: it is recommended that children spend between 80 and 120 min a day outdoors to reduce the progression and onset of myopia [6].
2. Pharmacological options: The application of atropine at low concentrations (between 0.01 and 0.05%) has shown good results in slowing the progression of myopia [7, 8].
3. Optical solution:
  - Contact lenses:
    Dual focus and multifocal: these include a variety of contact lens designs that generally feature a central zone for distance vision and concentric rings or a positive power gradient design that increases toward the periphery of the lens [9–12].
    Orthokeratology (OK): the use of OK lenses, designed for night use to flatten the central cornea, causes an elevation of the corneal mid-periphery, which causes a peripheral myopic blurring that helps control the axial growth of the eye [13, 14].
  - Ophthalmic lenses:
    Progressive and bifocal lenses: conflicting results and clinical evidence have especially been observed in the first year of such clinical trials [15–17].
    Peripheral defocus lenses: conflicting results and clinical evidence have especially been observed for a reduction of progression of the spherical equivalent refractive error [18–21].
    Simultaneous competing defocus: long-term clinical evidence with reductions of progression of both spherical equivalent refractive error and axial length [22, 23].

Of all the above-described options, ophthalmic lenses are the least invasive treatment to control myopia, so further research into new designs that improve current results should be a priority.

### Objective {7}

The objective is to set up a randomized, controlled, double-blind, and multicenter clinical trial to investigate the clinical evidence of a new design of ophthalmic lenses to reduce the progression of myopia.

### Trial design {8}

The trial design is a prospective, multicenter, randomized controlled, and double-blind clinical trial aimed to evaluate the efficacy of a new design of ophthalmic lenses to slow the progression of myopia in children.

## Methods: participants, interventions and outcomes

### Study setting {9}

**Fig. 1:**
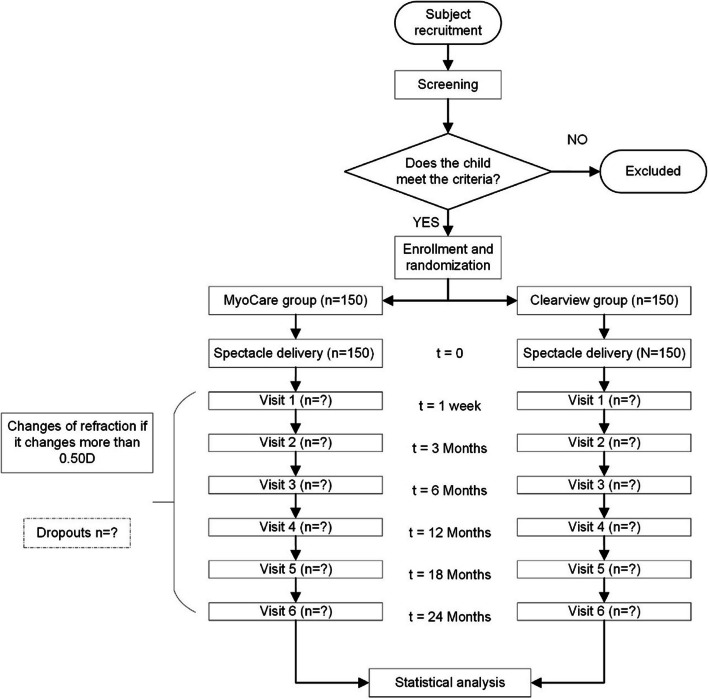
Study flowchart

The clinical trial is being done in the following ophthalmologic clinics in Spain and Portugal: Novovision (Madrid and Murcia, Spain), Virgen de Lujan clinic (Seville, Spain), Instituto clínico quirúrgico de Oftalmología—ICQO (Bilbao, Spain), Instituto de Microcirugía Ocular—IMO (Madrid, Spain), and Clínica Privada de Oftalmologia – CPO (Lisbon, Portugal). The trial is being coordinated by Universidad Complutense de Madrid and Instituto Superior de Educação e Ciências, ISEC Lisboa. Ethical approval was obtained from the Ethics Committee CEIm Hospital Clínico San Carlos. Written informed consent was collected from the parents/guardians and participants older than 12 years old before the enrolment. Table 1 shows the schedule of the different protocol phases: enrollment, follow-up visits, data collection, and assessment that were designed following the Standardized Protocol Items: Recommendations for Interventional Trials (SPIRIT) guidelines. The study flowchart is shown in Fig. 1.

**Table 1:** Schedule of assessments and examination items

Date/item		Screening	Spectacles dispense	Follow-up					
		Day − 30 to day 0	V0	V1	V2	V3	V4	V5	V6
			Day 0	1 week ± 1 day	3 months ± 15 days	6 months ± 30 days	12 months ± 30 days	18 months ± 30 days	24 months ± 30 days
Informed consent signed		X							
Basic information	Demographics	X							
	History	X							
Refraction	Noncycloplegic autorefraction	X				X	X	X	X
	Subjective refraction	X				X	X	X	X
	Cycloplegic autorefraction	X				X	X	X	X
Visual acuity	Uncorrected visual acuity	X				X	X	X	X
	Best-corrected visual acuity	X				X	X	X	X
	Central visual acuity		X		X	X	X	X	X
	Peripheral visual acuity		X		X				
Eye examination	Keratometry	X				X	X	X	X
	Slit lamp examination	X				X	X	X	X
	Fundus examination	X							
	Intraocular pressure measurement	X							
	Axial length	X			X	X	X	X	X
	Choroidal thickness	X				X	X	X	X
Binocular vision	Accommodative LAG	X				X	X	X	X
	Monocular estimated method (MEM) retinoscopy	X				X	X	X	X
	Phoria (distance and near)	X				X	X	X	X
Spectacle fitting	Frame position and lens condition		X						
Questionnaires	Lifestyle	X							
	Spectacle lens performance and wearability		X	X	X

### Eligibility criteria {10}

The study group will be made up of children aged from 6 to 13 (at the time of inclusion in the study) with a historical annual progression of spherical equivalent refractive error of − 0.50D.

The following are the inclusion criteria:

- Age 6–13 years
- Caucasian ethnicity
- Monocular/binocular decimal visual acuity with correction ≥ 1.00
- Spherical equivalent between − 0.75D and − 5.00D
- Astigmatism≤ 1.50D
- Anisometropia ≤1.00D
- Evolution of at least − 0.50D in 1 year

The following are the exclusion criteria:

- Presence of ocular pathology or a history of ocular and systemic abnormalities
- Strabismus or binocular vision problems
- History of eye surgery
- Having previously used some method to control myopia
- Having contraindications for the application of drugs for cycloplegia/corneal anesthesia

### Who will take informed consent? {26a}

Written informed consent was collected from the parents/guardians in the clinics before the screening visit. Participants older than 12 years old will sign also the informed consent adapted to their educational level.

### Additional consent provisions for collection and use of participant data and biological specimens {26b}

N/A.

### Interventions

#### Explanation for the choice of comparators {6b}

Optical design solutions such as dual focus/multifocal contact lenses and OK lenses based on the concept of simultaneous competing defocus have scientific evidence to reduce the progression of myopia in terms of SE and AxL [11, 13, 14, 24, 25]. This evidence-based knowledge from scientific concepts was translated into the lens design of ophthalmic lenses by taking into consideration the roughing eye. Applying simultaneous competing defocus due to cylindrical annular refractive elements, the eye experiences conflicting visual signals between the fovea and the periphery [23, 26]. These elements adopt optical microstructures with alternating defocus and correction zones, correcting myopia along the visual axis and simultaneously imposing relative myopic defocus over a large part of the retina for myopia progression management. Additionally, the back surface of the lenses is further supported by freeform optimization to correct the eye’s refractive error for all viewing angles and to minimize unintended hyperopic defocus for oblique views through the lens periphery.

#### Intervention description {11a}

Children assigned to the study group will wear ZEISS MyoCare lenses, while those assigned to the control group will wear conventional ZEISS ClearView single-vision lenses in their spectacles. Both lenses will be made of the same material (MR8) and hard as well as anti-reflection coating (ZEISS DuraVision Platinum) and will have the same surface treatments.

The following is the general overview:

- Subjective refraction (endpoint: best visual acuity with the most positive sphere).
- Objective refraction after cycloplegia (mean of 3 measurements).
- Ocular biometry (five measurements using IOLMaster 700 (Carl Zeiss Meditec AG, Jena, Germany)).
- OCT: spectral domain OCT using volume scan (and enhanced depth imaging) to visualize the choroid (ZEISS CIRRUS 5000, Carl Zeiss Meditec AG, Jena, Germany or Spectralis de Heildebergd Engineering) for 6 × 6 mm^2^ area. The choroidal thickness will be evaluated by automatic segmentation.
- Slit lamp examination.
- Intraocular pressure.
- Fundus check.
- Parameters of wear.
- Monocular measurement of distance and near visual acuity.
- Peripheral visual acuity.
- Distance phoria, near phoria @ 33 cm, and accommodative lag @ 33 cm.
- Vision quality, visual comfort, quality of life, and frequency of visual symptoms with lens wear are to be graded by the subjects themselves through questionnaires.

Table 1 describes the different measurements taken in each of the visits.

#### Criteria for discontinuing or modifying allocated interventions {11b}

As far as ophthalmic lenses are a non-invasive treatment, the only criterion for discontinuing the study will be that participants are not able to adapt to the design of the lenses (either control or intervention spectacle lenses). However, any participant is free to leave the study without any reason.

#### Strategies to improve adherence to interventions {11c}

An external CRO has created the electronic case report form (eCRF) and is responsible for giving access with different permissions to all the researchers and clinicians involved in the study. They also monitor the data included in the eCRF and ask the clinician for modifications or explanations when needed.

All the clinics will be also monitored in the different visits by an internal committee from Universidad Complutense de Madrid and ISEC Lisboa to ensure they adhere to intervention protocols.

#### Relevant concomitant care permitted or prohibited during the trial {11d}

There are no restrictions regarding concomitant care during the trial.

#### Provisions for post-trial care {30}

It is highly recommended that children with myopia have follow-ups every year to know the evolution and to check if spectacles need to be replaced. Children can follow up with their visits to the clinics and opticians that are going to participate in this clinical trial.

### Outcomes {12}

#### Primary outcome

The primary outcome is to compare the mean annual progression of the spherical equivalent between myopic children treated with ZEISS MyoCare lenses and those who wear a conventional single-vision lens.

#### Secondary outcomes

The following are the secondary outcomes:

1. Compare the mean annual progression of axial length between myopic children treated with ZEISS MyoCare lenses and those who wear a conventional single-vision lens
2. Compare the proportion of children in which the annual increase in the spherical equivalent does not exceed 0.5D in each of the groups (treatment and control)
3. Compare the proportion of children in which the annual increase in axial length does not exceed 0.22 mm in each of the groups (treatment and control)
4. Analyze twice a year the progression of myopia by studying the changes in the spherical equivalent, the axial length, the thickness of the choroid, the phorias, and the accommodative delay of both groups (control and treatment)
5. Evaluate the progression of the axial length and the spherical equivalent according to the age and graduation of the children at the beginning of the treatment in both groups (control and treatment)

### Participant timeline {13}

Table 1 shows the schedule of the different protocol phases: enrollment, follow-up visits, data collection, and assessment that were designed following the Standardized Protocol Items: Recommendations for Interventional Trials (SPIRIT) guidelines. The study flowchart is shown in Fig. 1.

### Sample size {14}

Based on an annual progression of − 0.50D, an estimated minimum efficacy of 50% compared to control, a power of 80%, an alpha of 5%, and a standard deviation for objective refraction of 0.70D, the size of the sample is 124.

Taking possible dropouts into account, 150 children will be included in the intervention group and 150 children in the control group.

Assignment to the different groups will be made by stratified randomization based on the age and spherical equivalent of the child. Randomization will be done automatically at the patient level by including the parameters of the initial visit in the eCRF. The researchers from the clinics will have partial access to the eCRF so that they will only be able to access the data that they have entered or must complete, not knowing the group assigned for each child.

### Recruitment {15}

For the recruitment of children, data from the myopia prevalence studies carried out by this research group since 2016 will be used. Since 2016, more than 12,000 children between 5 and 7 years of age have been screened, with a 17% prevalence of myopia [27, 28]. Children who have progressive myopia and have not been previously treated with any treatment to control myopia from the participating clinics, as well as those myopes followed up in the school’s screenings that we made annually in Lisbon, will be also included.

### Assignment of interventions: allocation

#### Sequence generation {16a}

The randomization has been programmed with minimization using 3 study variables for group stratification of sex, age group, and objective refraction group (spherical equivalent):

- Sex (2 options): male/female
- Age (2 options): 6–9 years/10–13 years
- Spherical equivalent (6 options): [− 0.5, − 1.25], [− 1.25, − 2], [− 2, − 2.75], [− 2.75, − 3.5], [− 3.5, − 4.25], [− 4.25, − 5]

The values will be computer-generated and assigned to the patients as A for the control group and B for the study lenses, with a 1:1 ratio.

#### Concealment mechanism {16b}

The allocation sequence will be sequentially numbered, independently if the children were assigned to the control or study group.

#### Implementation {16c}

All the children will pass the screening visit to assure that they comply with the inclusion criteria. Once the eCRF for the screening visit was filled in fully, the researcher from the clinic will use the specific option of the eCRF to generate the allocation sequence number. The children will be automatically assigned to the control or intervention group, but only the principal investigator could access this information.

### Assignment of interventions: blinding

#### Who will be blinded {17a}

The eCRF has different access depending on the role of the investigator. Only the PI and coordinator team members can access the information about the method of correction assigned to each child.

The spectacles will be fitted in opticians’ shops that do not participate in the follow-up visits, so the study is double-blinded for the investigators of the clinics and the participants.

#### Procedure for unblinding if needed {17b}

We do not anticipate any requirement for unblinding, but if required, the PI and coordinator team will have access to the group allocations and any unblinding will be reported.

### Data collection and management

#### Plans for assessment and collection of outcomes {18a}

Before starting the clinical trial, all researchers from each site have been trained in the protocol and data management. A CRO will be responsible for the data management and the elaboration of the eCRF where researchers can access the last version of the protocol.

Before the inclusion of the first patient, the users will be trained through the CRO training platform. In this training, the use of the eCRF application for data capturing will be explained through slides. Users will have to confirm that they understood the system and the characteristics of the studies by passing a test. Once the user has the certificate, he/she will receive the credentials to access the eCRF.

The training will be supported by the document “User Guide” which includes all the instructions for the correct use of the platform. The users can find this guide in the section “Documents” on the platform.

The investigator and the study team will also be provided with a CRO contact for any questions or problems related to the system.

Regarding the data entry, as eCRF, it will be done directly by the users. After the capture of the data, pages must be locked, thus allowing the data manager (DM) to begin the data review.

Only the principal investigator will be able to validate the data captured in the eCRF by electronically signing the eCRF pages.

The DM of the CRO will execute a biweekly validation of the data will detect the errors that were not rectified during the process of data capture and data monitoring and will issue an online query to the investigator to solve the discrepancy.

#### Plans to promote participant retention and complete follow-up {18b}

To promote participant retention:

1. All participants have been assigned to one investigator who is in contact with the participants and their parents during the whole study, solving any problems (broken spectacles, blurred vision, etc.).
2. In addition, annual reports will be done for parents to know the evolution of myopia.
3. Parents are also informed that all the children belonging to the control group that completes the 2-year study will be offered a spectacle to control myopia.

#### Data management {19}

The data management team from the CRO is specialized in capture, treatment, and data communication besides the designed processes for data management. This system will be used in the patient log, data capture, validation, query management, data coding, and extraction.

All documentation related to the database build and its validation will be kept in the data management study file (DMSF).

The clinical data management system (CDMS) will be built by the CRO using the ENNOV Capture System. ENNOV Capture System complies with RGPD UE Regulations regarding IT systems used in clinical trials, electronic signature, ICH E6 (R2), and FDA requirements.

#### Confidentiality {27}

Access to the eCRF is restricted by user password and role permission access. Logins and passwords are used to protect against unauthorized access to the database. The first time that the user connects to the platform, the system will request a password change. The password must have at least 8 characters.

Each user will be assigned to a pre-defined role (for example, CRA, data manager, investigator, read-only access) based on a job function that will determine access to specific tasks.

User accounts will be created by the CRO using the CSAdmin module or CSOnline, as appropriate. The DM will activate each new user after completing online training. The DM will ensure each role is enforced within the system and will send a confirmation email with the access data to the requester.

Permissions are personal and non-transferable. Permissions will be revoked upon notification of user termination, changes in project assignments, or study completion.

All medical data passing through the Internet network between browsers and the web server are encrypted with a 128-bit SSL certificate. In the case of computer hacking, medical data cannot be unveiled.

The activation of the security certificate can be verified by clicking the padlock in the web browser URL.

After 2 min of inactivity, the last data entered is automatically saved.

Other security information to take into account:

- Duration of password validity (months): 30 days by default (can be changed if necessary)
- Account expiration date: none by default (it is possible to define an expiration date)
- Automatic logout after a period of inactivity: 20 min by default (it cannot be changed)
- Account blocking: after 5 unsuccessful attempts to login

Each entry in the electronic audit trail will contain the date and time of action, author, data entry number, old value, new value, type of action (addition, modification, or correction), and the reason for the modification. All data captured in the eCRF will be collected in the audit trail.

#### Plans for collection, laboratory evaluation and storage of biological specimens for genetic or molecular analysis in this trial/future use {33}

Na.

Biological specimens have not been used in this clinical trial.

## Statistical methods

Data transfers will be sent to the PI from the CRO in SAS format and in Excel, and all databases will be included in one table per page. The data will be transferred by email using a compressed file (.zip) protected with a password. The database will be accompanied by the annotated eCRF (eCRF with the name of each variable impressed in it) to correctly locate each variable.

A validation will be performed before the final data transfer. This validation will include the following:

- A structure review to ensure the veracity
- A manual review of five records between the database and the files to assure that the data was correctly exported
- A review of the variables that appear in each table
- A verification of the total records of each table

### Statistical methods for primary and secondary outcomes {20a}

The statistical analysis of this study will be made by a statistician who will participate in the entire process of study design, implementation, data management, analysis of study results, and summary. Once the study protocol and CRF are completed, the statistical analysis plan will be finalized before the study data is locked to complete the statistical analysis report.

Statistical analysis will be performed using Stata. The statistical description and inference of the data will be based on the characteristics of the data and the selection of applicable descriptive indicators and hypothesis testing methods.

Efficacy analyses will be performed based on the full analysis set and per the protocol set. All baseline demographic analyses will be performed based on the analysis set.

The data included will be analyzed using the applicable statistical methods according to their distribution characteristics. Unless otherwise specified, quantitative endpoints will be described by calculating the mean, standard deviation, median, minimum, maximum, lower quartile (Q1), and upper quartile (Q3), and the categorical assessments will be described by calculating the number and percentage of subjects. Quantitative endpoint cluster comparisons will be performed using the cluster *t*-test, or Wilcoxon rank sum test depending on the data distribution, and categorical endpoint cluster comparisons will be performed using the chi test—square or the exact probability test.

For studies of the evolution of the progression of myopia, ANOVAs of repeated measures or Kruskal–Wallis tests will be performed.

Finally, to study how the age and graduation of the child at the start of treatment are affected, a linear regression model will be built to assess the annual change in myopia and axial length analyzing data from the right eyes of all children, what is a standard practice in this kind of studies [29–32].

All statistical tests will be determined to be statistically significant based on *P* < 0.05.

### Interim analyses {21b}

This study will have a 2-year duration, and it is not planned to do any interim analysis.

### Methods for additional analyses (e.g., subgroup analyses) {20b}

There is no plan to make any additional analyses.

### Methods in analysis to handle protocol non-adherence and any statistical methods to handle missing data {20c}

To minimize these problems, an “intention-to-treat analysis” will be done as the primary analysis, with a per-protocol analysis as a secondary/supplementary analysis.

**Fig. 2:**
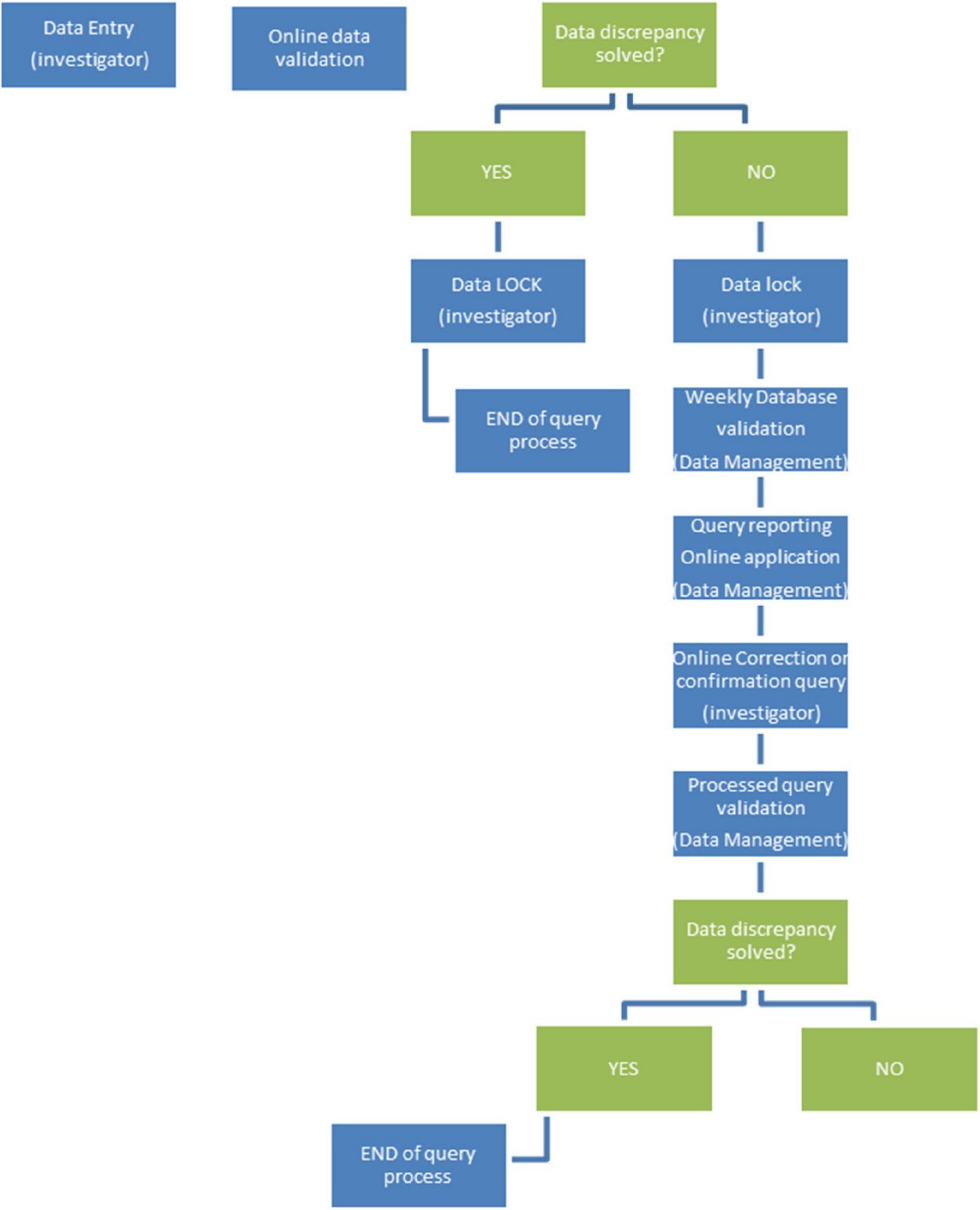
Data entry and validation flowchart

Data management includes the query management process made by the CRO. Data validation will be automatic during the data capture done by the investigator. Figure 2 shows the data entry and validation flowchart.

### Plans to give access to the full protocol, participant-level data and statistical code {31c}

The datasets analyzed during the current study and the statistical code are available from the corresponding author upon reasonable request, as is the full protocol.

### Oversight and monitoring

#### Composition of the coordinating center and trial steering committee {5d}

The trial steering committee is composed of three members of the funders and three from the sponsor, including the principal investigator. They receive biweekly information about how the study is running.

The coordinating center is composed of 3 people, including the principal investigator. They are the only ones that know all the information about the children, and they coordinate all the visits of the study. They are also the main contact with the children’s parents.

Regarding the statistician, she is not part of the steering committee, but she participated in the design of the clinical trial and in the elaboration of the CRF, and she will assess the steering committee during the whole clinical trial.

#### Composition of the data monitoring committee, its role and reporting structure {21a}

The members of the coordinating center monitoring are in the first visits of each part of the clinical trial (screening, 3-, 6-, 12-, and 18-month visits) to assure that the protocol has been followed and there is not any doubt about it.

They, together with the DM of the CRO, form the data monitoring committee. The DM of the CRO will execute a biweekly validation of the data to detect the errors that were not rectified during the process of data capture and data monitoring and will issue an online query to the investigator to solve the discrepancy.

#### Adverse event reporting and harms {22}

Adverse events will be registered in the eCRF and treated urgently by the members of the coordinating center that are in contact with all the investigators as well as the parents involved in the clinical trial.

#### Frequency and plans for auditing trial conduct evolution of the study {23}

In addition to the biweekly audits done by the CRO and the monitoring from the coordinating center members that monitor every center the first day of each visit (screening, 3, 6, 12, 18, and 24 months), this clinical trial will be audited by the external ethics committee, which will receive semestral reports about the evolution of the study.

#### Plans for communicating important protocol amendments to relevant parties (e.g., trial participants, ethical committees) {25}

Any change in the protocol will be communicated to the ethical committee, investigators, CRO, and participants by the coordinating center members by email and phone.

#### Dissemination plans {31a}

The dissemination of the results, given their clinical nature, must reach ophthalmologists and optometrists who deal with myopic children. We will participate and organize sessions at a local level, through schools or scientific societies in the area.

Likewise, the advances and results of the research will be communicated both in the main congresses of the specialty (Association for Research in Vision and Ophthalmology, International Myopia Conference, etc.) and in MEDLINE-registered journals.

## Discussion

The present trial was designed to investigate the efficacy of a new design of ophthalmic lenses for the management of myopia. The present study is the first randomized, controlled, double-blind, and multicenter trial that looks for the efficacy of ZEISS MyoCare spectacle lenses in Caucasian children in Europe.

Understanding, controlling, and treating myopia is an objective of the World Health Organization, through the Vision 2020 project [33]. According to estimates by the Global Burden of Disease, myopia is the second leading cause of blindness in the world [34]. At a global level, including developed and non-developed countries, it is responsible for 23% of blindness cases and 53% of moderately low vision or visual impairment cases [35].

This project, to the extent that it allows effective control of myopia with a non-invasive method, will help to reduce the magnitude of myopia in the population, and thus, morbidity will be significantly reduced.

The fulfillment of the objectives of this project will allow the implantation of a new design of ophthalmic lenses for the control of myopia.

## Trial status

At the time of submission, recruitment has started, and a third of the participants were under the 3 months follow-up visit. The recruitment started on 15 September 2022 and is expected to finish on 1 June 2023. A total of 300 children will be recruited, with 250 already enrolled. The last 24-month visit is expected to be completed on 31 July 2025. The protocol version is 3.0, dated 24 November 2022.

The manuscript was not submitted earlier because we did not know how many opticians were going to be involved in the cut, edge, and fit of the spectacles, and they should be included in the acknowledgments. The last subject has been enrolled on 25 May 2023, and the last visit is planned by the end of May 2025.

## Data Availability

The eCRF and study database records will be retained on the CRO server for 25 years after the completion of the study. Also, even though the database is locked, it will be accessible for the CRO and the sponsor, but no changes can be made.

## Abbreviations

OK: Orthokeratology
SPIRIT: Standardized Protocol Items: Recommendations for Interventional Trials guidelines
VA: Visual acuity
IOP: Intraocular pressure measurement
AL: Axial length
CRO: Clinical Research Organization
eCRF: Electronic case report form
PI: Principal investigator
DM: Data manager
DMSF: Data management study file
CDMS: Clinical data management system
